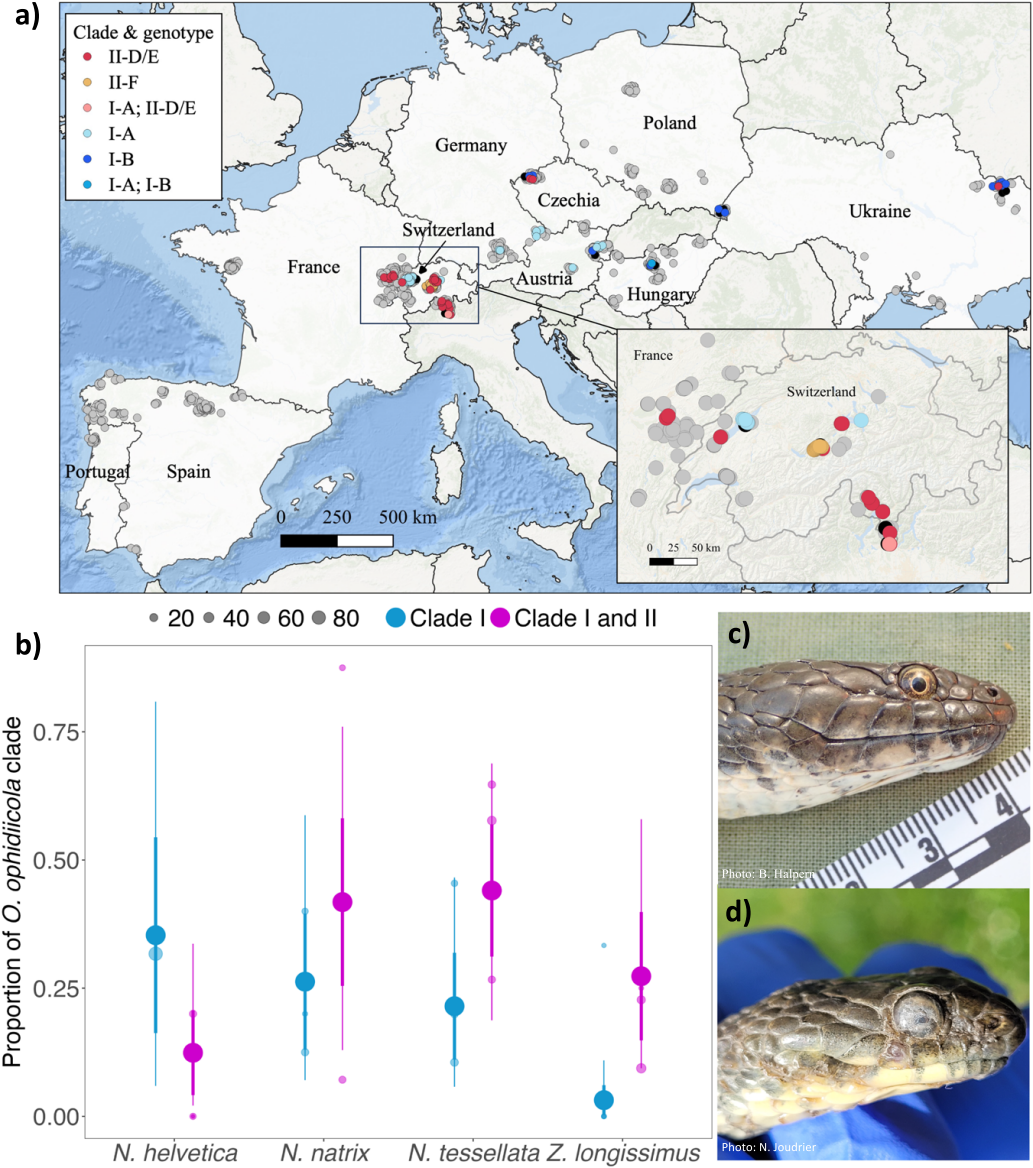# Author Correction: Contribution of host species and pathogen clade to snake fungal disease hotspots in Europe

**DOI:** 10.1038/s42003-024-06424-x

**Published:** 2024-06-25

**Authors:** Gaëlle Blanvillain, Jeffrey M. Lorch, Nicolas Joudrier, Stanislaw Bury, Thibault Cuenot, Michael Franzen, Fernando Martínez-Freiría, Gaëtan Guiller, Bálint Halpern, Aleksandra Kolanek, Katarzyna Kurek, Olivier Lourdais, Alix Michon, Radka Musilová, Silke Schweiger, Barbara Szulc, Sylvain Ursenbacher, Oleksandr Zinenko, Joseph R. Hoyt

**Affiliations:** 1 Biological Sciences Department, Virginia Polytechnic Institute and State University, Blacksburg, VA USA; 2 U.S. Geological Survey, National Wildlife Health Center, Madison, WI USA; 3 Institute of Biology, University of Neuchâtel, Neuchâtel, Switzerland; 4 Institute of Animal Pathology, University of Bern, Bern, Switzerland; 5 Info fauna-Karch, Centre Suisse de Cartographie de la Faune (CSCF) and Centre de coordination pour la protection des reptiles et des amphibiens de Suisse (karch), Neuchâtel, Switzerland; 6 Department of Comparative Anatomy, Institute of Zoology and Biomedical Research, Jagiellonian University, Cracow, Poland; 7 NATRIX Herpetological Association, Wroclaw, Poland; 8 LPO Bourgogne-Franche-Comté, Site de Franche-Comté, Maison de l’environnement de BFC, Besançon, France; 9 Bavarian State Collection of Zoology (ZSM-SNSB), Munich, Germany; 10 CIBIO, Centro de Investigação em Biodiversidade e Recursos Genéticos, InBIO Laboratório Associado, Campus de Vairão, University of Porto, Vairão, Portugal; 11 BIOPOLIS Program in Genomics, Biodiversity and Land Planning, CIBIO, Campus de Vairão, Vairão, Portugal; 12 Le Grand Momesson, Bouvron, France; 13 MME BirdLife Hungary, Budapest, Hungary; 14 Department of Systematic Zoology and Ecology, Institute of Biology, Eötvös Loránd University, Budapest, Hungary; 15 HUN-REN-ELTE-MTM, Integrative Ecology Research Group, Budapest, Hungary; 16 Department of Geoinformatics and Cartography, Institute of Geography and Regional Development, Faculty of Earth Sciences and Environmental Management, University of Wroclaw, Wroclaw, Poland; 17 Department of Wildlife Conservation, Institute of Nature Conservation Polish Academy of Science, Cracow, Poland; 18 Centre d’Etudes Biologiques de Chizé, ULR CNRS UMR 7372, Villiers en Bois, France; 19 School of Life Sciences, Arizona State University, Tempe, AZ USA; 20 Zamenis Civic Association, Karlovy Vary, Czech Republic; 21 First Zoological Department, Herpetological Collection, Natural History Museum, Vienna, Austria; 22 Department of Genetics, Kazimierz Wielki University, Bydgoszcz, Poland; 23 Department of Environmental Sciences, Section of Conservation Biology, University of Basel, Basel, Switzerland; 24 Balaton Limnological Research Institute, Tihany, Hungary; 25 V.N. Karazin Kharkiv National University, Kharkiv, Ukraine

**Keywords:** Ecological epidemiology, PCR-based techniques, Biogeography

Correction to: *Communications Biology* 10.1038/s42003-024-06092-x, published online 10 April 2024

The original version of the article contained errors in the maps presented in Figs. 1 and 4. These have now been corrected in the PDF and HTML versions of the Article.

Original figure:

Figure 1
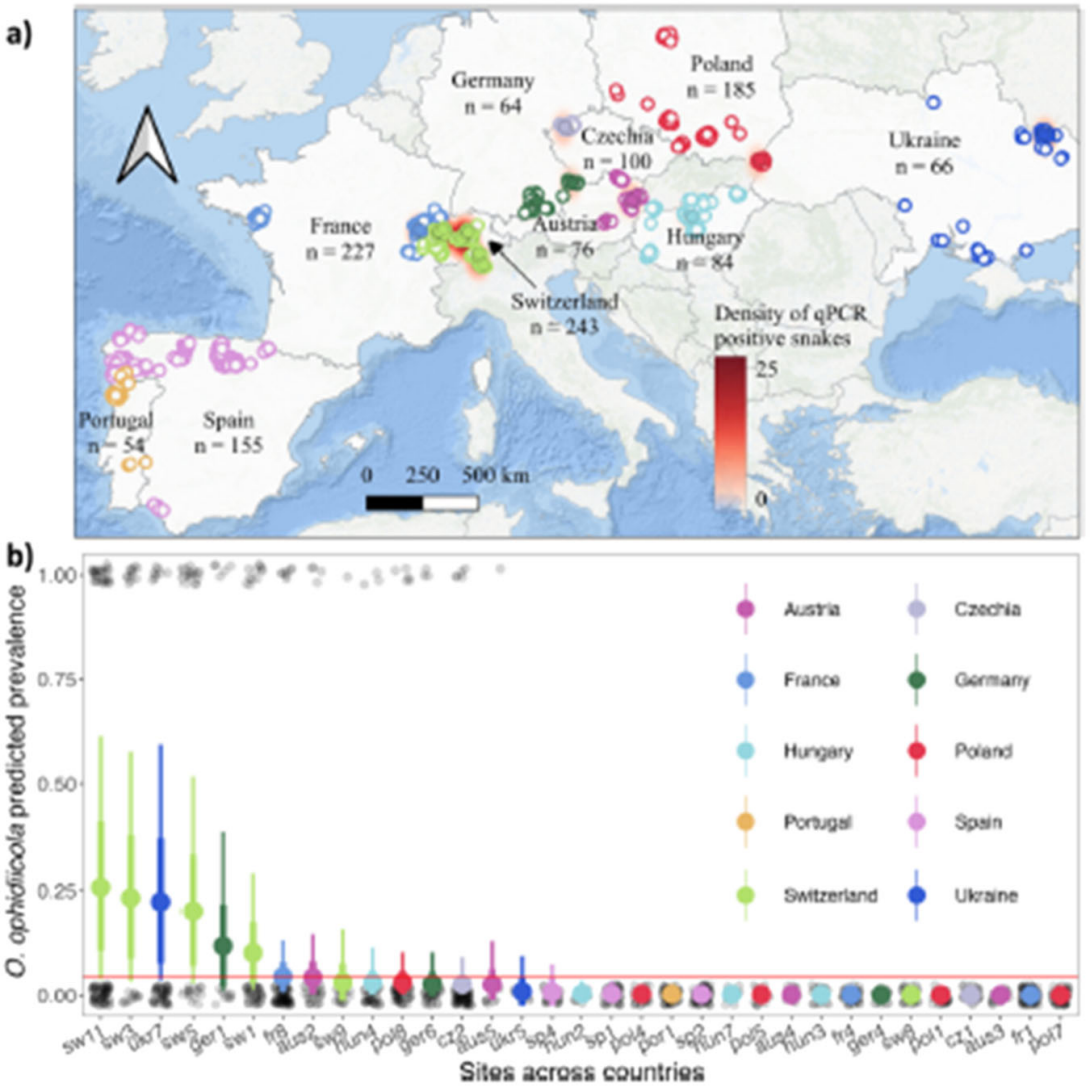


Figure 4
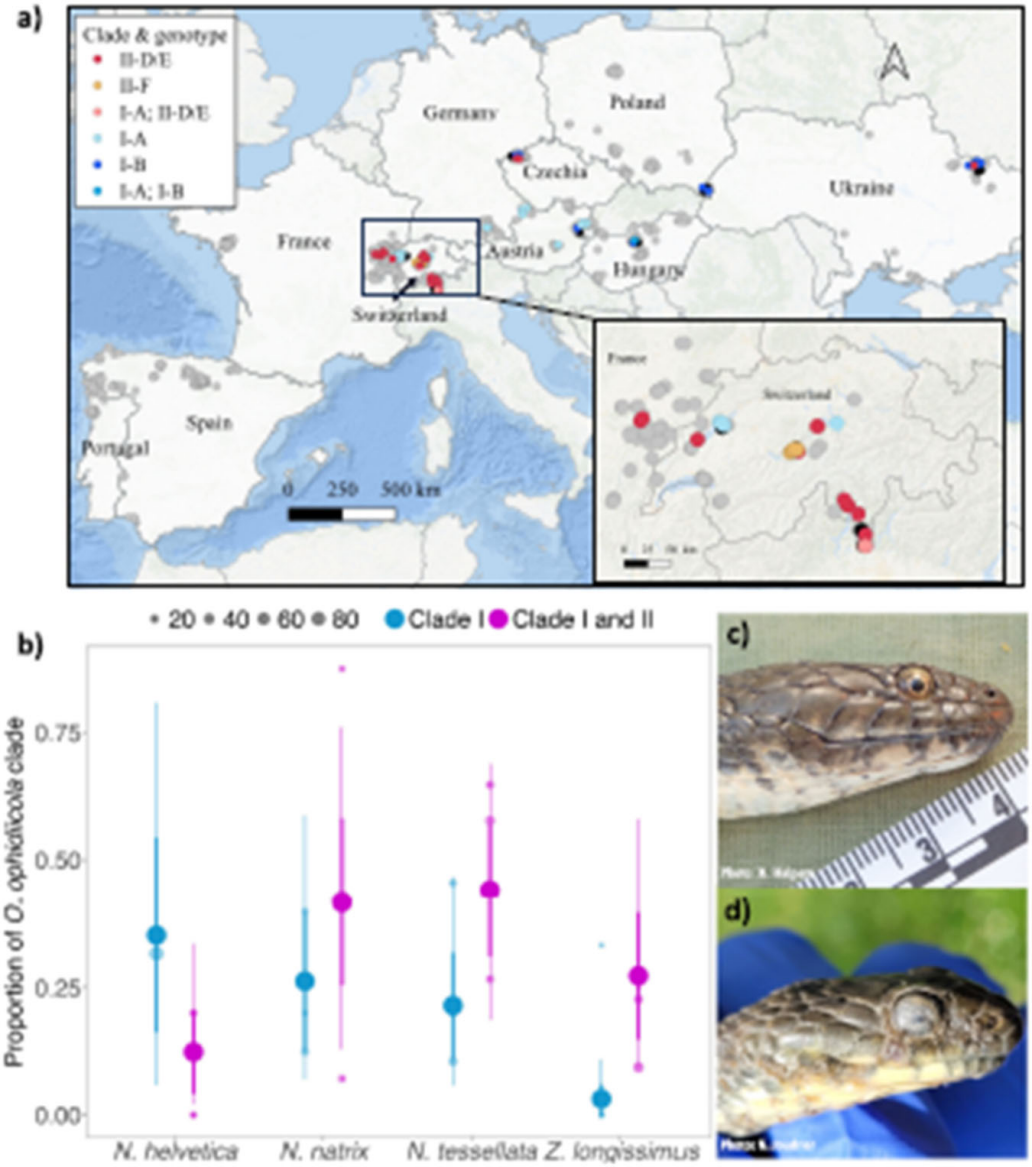


Corrected figure:

Figure 1
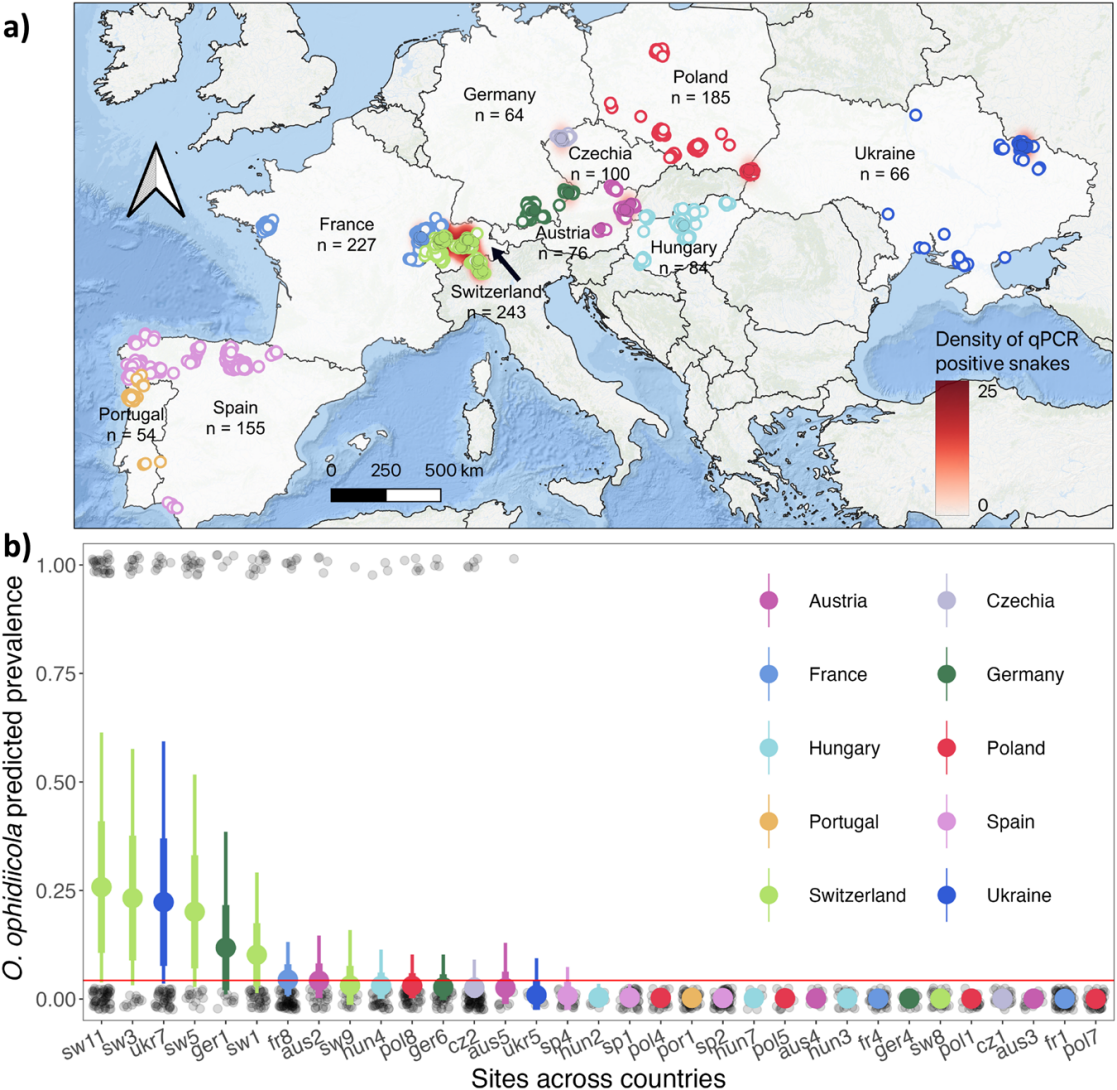


Figure 4