# Supplementary material for: Contribution of host species and pathogen clade to snake fungal disease hotspots in Europe
Source: Commun Biol. 2024 Apr 10;7:440. doi: 10.1038/s42003-024-06092-x (PMC11006896; doi:10.1038/s42003-024-06092-x)
Supplement: Supplementary file 2 — Supplementary information [file 42003_2024_6092_MOESM2_ESM.pdf]

Supporting information

Supplementary Table 1. Summary of all figures, tables and corresponding statistical analyses.

Figure	Table	Distribution	Response variable	Population-effect	Group-effect
S2	-	Bernoulli	Pathogen detection on snakes (0|1)	Julian date of each snake's capture date	Species + Site
1b	S3	Bernoulli	Pathogen detection on snakes (0|1)	Site	Species
N/A	N/A	Beta	Site prevalence	Average SVL for each snake species	Species
N/A	N/A	Bernoulli	Pathogen detection on snakes (0|1)	Genus	Site
2	S4	Bernoulli	Pathogen detection on snakes (0|1)	Species	Site
3a	-	Bernoulli	Lesion observed (0|1)	Species	Site
3b	S5	Beta	Proportion of total surface area of snake covered in lesion	Species	Site
4b	S7	Bernoulli	Pathogen detection on snakes (0|1)	Clade * Species	Site
S5	-	Beta	Proportion of total surface area of snake covered in lesion	Clade	Species

Supplementary Table 2. Description of sites, species and sample sizes across all 10 countries sampled from March 2020 to June 2022 for the presence of *Ophidiomyces ophidiicola*, the pathogen responsible for Snake Fungal Disease. The 4-letter codes represent genus and species for each snake species sampled as follows: *Coronella austriaca* (COAU), *Coronella girondica* (COGI), *Dolichophis caspius* (DOCA), *Elaphe sauromates* (ELSA), *Hierophis viridiflavus* (HIVI), *Malpolon monspessulanus* (MAMO), *Natrix astreptophora* (NAAS), *Natrix helvetica* (NAHE), *Natrix natrix* (NANA), *Natrix maura* (NAMA), *Natrix tessellata* (NATE), *Vipera ammodytes* (VIAM), *Vipera aspis* (VIAS), *Vipera berus* (VIBE), *Vipera latastei* (VILA), *Vipera nikolskii* (VINI), *Vipera renardi* (VIRE), *Vipera seoanei* (VISE), *Vipera ursini* (VIUR), *Zamenis longissimus* (ZALO), *Zamenis scalaris* (ZASC). For each species, the numbers within each cell represent: total number of snakes with skin lesion / total number of snakes testing positive by qPCR / total number of snakes sampled. The latitudinal and longitudinal ranges for each site are provided in decimal degrees.

AUSTRIA (n = 76)

Site	Latitudinal range	Longitudinal range	*Species*				
			COAU	NANA	NATE	VIAM	ZALO
Aus1	48.18-48.15	16.49-16.55	-	-	2/3/3	-	-
Aus2	48.04-47.80	16.08-16.22	0/0/2	-	0/4/20	0/0/7	0/0/5
Aus3	48.71-48.72	15.65-15.66	-	2/0/18	-	-	-
Aus4	48.15-48.14	16.68-16.70	1/0/1	1/0/8	-	-	1/0/3
Aus5	47.38-47.25	14.92-15.16	-	0/1/5	-	-	0/0/4

CZECH REP (n = 100)

Site	Latitudinal range	Longitudinal range	*Species*				
			COAU	NANA	NATE	VIBE	ZALO
Cz1	50.39-50.34	13.28-13.37	-	0/0/1	0/0/17	-	0/0/8
Cz2	50.43-50.32	12.82-13.08	4/1/19	2/1/14	-	0/0/9	3/3/32

FRANCE (n = 227)

Site	Latitudinal range	Longitudinal range	*Species*						
			COAU	HIVI	NAHE	NAMA	VIAS	VIBE	ZALO
Fr1	47.78-47.38	-1.87/-1.67	0/0/2	-	0/0/25	-	0/0/10	0/0/14	0/0/40
Fr2	47.77-47.59	6.39-6.76	0/0/1	-	-	-	-	-	-
Fr3	47.15-47.36	6.68-6.87	1/0/1	-	-	-	0/0/6	-	-
Fr4	47.32-47.27	6.12-6.27	-	0/0/6	0/0/4	1/0/17	-	-	2/0/17
Fr5	47.20-47.09	6.44-6.72	0/0/1	-	-	-	0/0/5	-	-
Fr6	46.61-46.47	5.70-6.02	0/0/1	-	-	-	-	-	-
Fr7	46.34-46.24	5.52-5.68	0/0/2	0/0/2	-	-	-	-	-
Fr8	47.25-46.81	5.65-6.24	4/0/8	3/2/10	1/0/10	0/0/8	0/0/5	0/0/10	8/5/22

GERMANY (n = 64)

Site	Latitudinal range	Longitudinal range	*Species*					
			COAU	NAHE	NANA	NATE	VIBE	ZALO
Ger1	48.66-48.49	13.39-13.76	-	-	-	4/5/11	-	2/0/3
Ger2	48.30-48.14	12.71-13.08	0/0/1	-	-	-	-	-
Ger3	47.84-47.61	11.98-12.47	-	2/0/3	-	-	0/0/1	-
Ger4	48.20-48.12	11.30-11.51	-	-	0/0/13	-	-	-
Ger5	47.73-47.58	11.08-11.30	-	-	-	-	0/0/1	-
Ger6	48.03-47.76	11.22-11.45	0/0/2	-	1/2/16	-	1/1/13	-

HUNGARY (n = 84)

Site	Latitudinal range	Longitudinal range	*Species*						
			COAU	DOCA	NANA	NATE	VIBE	VIUR	ZALO
Hun1	48.29-48.14	21.25-21.54	-	-	0/0/2	0/0/5	-	-	-
Hun2	48.09-47.99	18.85-19.09	0/0/3	0/0/1	0/0/3	-	-	-	0/0/1
Hun3	47.14-46.94	19.18-19.52	1/0/3	-	0/0/7	-	-	0/0/8	-
Hun4	47.60-47.41	18.96-19.31	0/0/1	1/0/11	-	4/2/19	-	-	0/1/3
Hun5	47.85-47.60	17.14-17.54	-	-	-	-	-	0/0/6	-
Hun6	47.42-47.20	17.07-17.41	-	-	-	-	0/0/1	-	-
Hun7	46.40-46.20	17.06-17.38	-	-	0/0/5	-	0/0/5	-	-

POLAND (n = 185)

Site	Latitudinal range	Longitudinal range	*Species*			
			COAU	NANA	NATE	ZALO
Pol1	53.15-52.95	17.78-18.20	2/0/12	0/0/15	-	-
Pol2	51.44-51.30	15.46-15.80	0/0/2	-	-	-
Pol3	51.25-51.05	16.86-17.25	-	1/0/1	-	-
Pol4	50.59-50.44	17.90-18.23	3/0/21	0/0/3	-	-
Pol5	49.87-49.72	18.43-18.78	-	-	0/0/11	-

Pol6	50.47-50.02	21.20-21.82	0/0/2	-	-	-
Pol7	50.11-49.95	19.77-20.12	11/0/21	7/0/46	-	-
Pol8	49.32-49.14	22.35-22.80	-	4/4/10	-	3/1/41

PORTUGAL (n = 54)

Site	Latitudinal range	Longitudinal range	*Species*					
			COAU	MAMO	NAAS	NAMA	VILA	VISE
Por1	42.05-41.28	-8.73/-8.12	1/0/4	0/0/1	0/0/5	0/0/1	4/0/34	0/0/6
Por2	38.87-38.68	-7.30/-7.01	-	-	-	0/0/3	-	-

SPAIN (n = 155)

Site	Latitudinal range	Longitudinal range	*Species*								
			COAU	COGI	MAMO	NAAS	NAMA	VIAS	VILA	VISE	ZASC
Sp1	43.70-41.98	-8.86/-7.27	0/0/4	-	-	0/0/7	0/0/4	-	-	2/0/26	-
Sp2	43.07-42.17	-6.72/-3.33	0/0/1	0/0/1	-	1/0/2	0/0/1	1/0/23	0/0/29	0/0/19	-
Sp3	37.26-36.82	-6.95/-6.26	-	0/0/1	-	-	-	-	-	-	1/0/3
Sp4	42.49-42.11	-2.98/-2.21	0/0/1	0/0/1	0/0/1	-	0/0/2	1/0/11	0/0/12	-	-
Sp5	43.03-42.83	-1.73/-1.39	-	-	0/0/1	0/0/1	-	0/0/4	-	-	-

SWITZERLAND (n = 243)

Site	Latitudinal range	Longitudinal range	*Species*							
			COAU	HIVI	NAHE	NAMA	NATE	VIAS	VIBE	ZALO
Sw1	46.28-45.84	8.53-8.98	-	2/3/11	4/3/15	-	3/4/15	-	-	2/1/4
Sw2	46.99-47.21	8.57-8.80	-	-	-	1/1/1	0/0/1	-	-	-
Sw3	46.98-46.94	8.27-8.33	0/0/2	-	0/0/1	-	11/11/17	0/0/2	-	-
Sw4	46.79-46.68	8.19-8.36	-	-	0/0/3	-	-	0/0/1	0/0/3	-
Sw5	46.75-46.67	7.89-8.07	-	-	0/0/2	-	14/15/26	0/0/1	-	-
Sw6	46.36-46.20	6.87-7.23	-	1/0/6	-	-	-	-	-	-
Sw7	46.50-46.39	6.12-6.29	-	0/0/2	-	-	-	-	-	-

Sw8	46.55-46.44	6.66-6.92	-	1/0/8	-	0/0/2	11/0/16	-	-	-
Sw9	46.90-46.74	6.53-6.75	-	1/1/13	-	-	-	0/0/4	-	-
Sw10	47.03-46.94	6.67-6.85	-	-	-	-	-	-	0/0/4	-
Sw11	47.04-46.95	6.95-7.11	-	0/0/1	26/26/82	-	-	-	-	-

UKRAINE (n = 66)			*Species*							
Site	Latitudinal range	Longitudinal range	COAU	DOCA	ELSA	NANA	NATE	VIBE	VINI	VIRE
Ukr1	51.22-51.02	30.58-30.98	-	-	-	-	-	0/0/1	-	-
Ukr2	47.89-47.73	29.30-29.59	0/0/1	-	-	-	-	-	-	-
Ukr3	46.72-46.60	31.03-31.27	-	0/0/2	-	-	-	-	-	-
Ukr4	47.26-47.06	31.89-32.14	-	0/0/1	-	-	-	-	-	-
Ukr5	46.37-46.07	33.13-33.49	-	0/0/1	0/0/3	0/0/1	-	-	-	0/0/6
Ukr6	46.48-46.31	35.10-35.37	-	-	-	-	0/0/1	-	-	-
Ukr7	50.18-49.29	35.12-37.09	0/0/3	-	-	7/7/8	-	-	9/1/32	0/0/6

Supplementary Table 3. Coefficients from Bayesian hierarchical model for species-level *Ophidiomyces ophidiicola* prevalence, the pathogen that causes snake fungal disease, in Europe (coefficient ± standard deviation (95% credible intervals)). The data were analyzed with a Bernoulli distribution and logit link including site as a population-level effect and species as a random effect. The model is parameterized in relation to the reference level or intercept site *fr8*, and each parameter represents the difference from this reference level. Exact location of sites is included in Table S2.

	Estimate ± Standard Deviation (95% CI)
Intercept, Site fr8	-3.36±0.82 (-5.12,-1.92)
Site sw1	0.91±0.66 (-0.38,2.19)
Site sw11	2.13±0.78 (0.68,3.74)
Site aus2	-0.14±0.85 (-1.87,1.46)
Site aus3	-9.1±5.46 (-22.54,-1.76)
Site aus4	-8.8±5.65 (-22.75,-1.06)
Site aus5	-1.12±1.42 (-4.34,1.32)
Site cz1	-9.06±5.61 (-22.65,-1.48)
Site cz2	-0.62±0.75 (-2.08,0.83)
Site fr1	-8.78±5.43 (-22.53,-1.79)
Site fr4	-8.54±5.39 (-21.43,-1.24)
Site ger1	1.01±0.85 (-0.71,2.6)
Site ger4	-8.98±5.59 (-22.49,-1.46)
Site ger6	-0.71±0.94 (-2.6,1.11)
Site hun2	-8.37±6.16 (-23.11,-0.08)
Site hun3	-8.58±5.69 (-22.08,-1)
Site hun4	-0.52±0.92 (-2.41,1.23)
Site hun7	-8.55±5.9 (-22.85,-0.52)
Site pol1	-9.1±5.69 (-23.02,-1.62)
Site pol4	-8.17±5.79 (-22.13,-0.16)

Site pol5	-8.64±5.73 (-22.02,-0.82)
Site pol7	-9.43±5.27 (-22.47,-2.61)
Site pol8	-0.47±0.74 (-1.98,0.96)
Site por1	-8.08±5.68 (-21.8,-0.22)
Site sp1	-8.17±5.76 (-21.97,-0.07)
Site sp2	-8.41±5.77 (-22.02,-0.28)
Site sp4	-7.68±5.98 (-21.82,0.9)
Site sw3	1.95±0.8 (0.43,3.59)
Site sw5	1.76±0.76 (0.28,3.28)
Site sw8	-8.9±5.57 (-22.37,-1.49)
Site sw9	-0.96±1.43 (-4.32,1.37)
Site ukr5	-7.63±6.41 (-22.88,1.33)
Site ukr7	1.88±0.95 (0.14,3.84)

Supplementary Table 4. Coefficients from Bayesian hierarchical model for species-level *Ophidiomyces ophidiicola* prevalence, the pathogen that causes snake fungal disease, in Europe (coefficient ± standard deviation (95% credible intervals)). The data were analyzed with a Bernoulli distribution and logit link including species as a population-level effect and site as a random effect (*Coronella austriaca* (COAU), *Dolichophis caspius* (DOCA), *Hierophis viridiflavus* (HIVI), *Natrix astreptophora* (NAAS), *Natrix helvetica* (NAHE), *Natrix natrix* (NANA), *Natrix maura* (NAMA), *Natrix tessellata* (NATE), *Vipera aspis* (VIAS), *Vipera berus* (VIBE), *Vipera latastei* (VILA), *Vipera nikolskii* (VINI), *Vipera renardi* (VIRE), *Vipera seoanei* (VISE), *Vipera ursini* (VIUR), *Zamenis longissimus* (ZALO)). The model is parameterized in relation to the reference level or intercept NATE, and each parameter represents the difference from this reference level.

	Estimate ± Standard Deviation (95% CI)
Intercept (NATE)	-2.8±0.8 (-4.6,-1.42)
Species COAU	-3.69±1.37 (-6.73,-1.43)
Species DOCA	-8.24±5.76 (-21.88,-0.49)
Species HIVI	-0.65±0.66 (-2,0.62)
Species NAAS	-7.93±6.02 (-22.23,0.8)
Species NAHE	-1.33±0.66 (-2.69,-0.1)
Species NAMA	-8.79±5.42 (-22.1,-1.4)
Species NANA	-0.17±0.78 (-1.72,1.39)
Species VIAS	-9.31±5.14 (-21.37,-2.21)
Species VIBE	-2.66±1.34 (-5.53,-0.4)
Species VILA	-8.69±5.5 (-21.92,-0.67)
Species VINI	-5.14±1.62 (-8.82,-2.3)
Species VIRE	-10.19±5.62 (-23.64,-2.52)
Species VISE	-8.43±5.54 (-21.45,-0.2)
Species VIUR	-6.63±6.31 (-20.54,3.33)
Species ZALO	-1.01±0.63 (-2.27,0.14)

Supplementary Table 5. Coefficients from Bayesian hierarchical model for differences in severity of snake fungal disease (SFD) among species in Europe for individuals that were confirmed positive for the pathogen that causes SFD, *Ophidiomyces ophidiicola,* through quantitative polymerase chain reaction (qPCR) (coefficient ± standard deviation (95% credible intervals)). The data were analyzed with a beta distribution that included site as random effect, and species as a population-level effect. The model is parameterized in relation to the reference level or intercept *Natrix tessellata* and each parameter represents the difference from this reference level.

	Estimate ± Standard Deviation (95% CI)
Intercept *N. tessellata*	-5.43±0.26 (-5.88,-4.8)
species *Z. longissimus*	0.03±0.46 (-0.85,0.98)
species *N. helvetica*	-0.29±0.42 (-1.25,0.54)
species *N. natrix*	0.32±0.55 (-1.07,1.06)
species *C. austriaca*	0.23±1.03 (-2.3,1.71)
species *H. viridiflavus*	-0.07±0.5 (-1.14,0.82)

Supplementary Table 6. Alignment of representative sequences previously deposited in GenBank that correspond to the four internal transcribed spacer 2 (ITS2) region genotypes detected in our study.

```
Genotype_IB_KY474061         GAAATGCGATAAGTAATGTGAATTGCAGAATTCCGTGAATCATCGAATCTTTGAACGCAC     60
Genotype_IIF_KX148658.1      GAAATGCGATAAGTAATGTGAATTGCAGAATTCCGTGAATCATCGAATCTTTGAACGCAC     60
Genotype_IA_KY474059.1       GAAATGCGATAAGTAATGTGAATTGCAGAATTCCGTGAATCATCGAATCTTTGAACGCAC     60
Genotype_IIDE_OL457490.1     GAAATGCGATAAGTAATGTGAATTGCAGAATTCCGTGAATCATCGAATCTTTGAACGCAC     60
                             ************************************************************

Genotype_IB_KY474061         ATTGCGCCCCCTGGTATTCCGGGGGGCATGCCTGTCCGAGCGTCATTGCAACCCCCTCAA     120
Genotype_IIF_KX148658.1      ATTGCGCCCCCTGGTATTCCGGGGGGCATGCCTGTCCGAGCGTCATTGCAACCCCCTCAA     120
Genotype_IA_KY474059.1       ATTGCGCCCCCTGGTATTCCGGGGGGCATGCCTGTCCGAGCGTCATTGCAACCCCCTCAA     120
Genotype_IIDE_OL457490.1     ATTGCGCCCCCTGGTATTCCGGGGGGCATGCCTGTCCGAGCGTCATTGCAACCCCCTCAA     120
                             ************************************************************

Genotype_IB_KY474061         GCCCGGCTTGTGTGTTGGGGGCGCCCGCCCCGAAGTCCTCGGGCGCGGGCCCCCCCCCAA     180
Genotype_IIF_KX148658.1      GCCCGGCTTGTGTGTTGGGGGCGCCCACCCCGAAGTCCTCGGGCGCGGGCCCCCCCCCAA     180
Genotype_IA_KY474059.1       GCCCGGCTTGTGTGTTGGGGGCGCCCGCCCCGAAGTCCTCGGGCGCGGGCCC-CCCCCAA     179
Genotype_IIDE_OL457490.1     GCCCGGCTTGTGTGTTGGGGGTGCCCACCCCGAAGTCCTCGGGCGCGGGCCC-CCCCCAA     179
                             ********************* **** ************************* *******

Genotype_IB_KY474061         ATGCAGTGGCGGCACCGAGTTCCTGGTGTCTGAGTGTATGGGAATCTGTTTCTGTCTCGC     240
Genotype_IIF_KX148658.1      ATGCAGTGGCGGCACCGAGTTCCTGGTGTCTGAGTGTATGGGAATCTGTTTCTGTCTCGC     240
Genotype_IA_KY474059.1       ATGCAGTGGCGGCACCGAGTTCCTGGTGTCTGAGTGTATGGGAATCTGTTTCTGTCTCGC     239
Genotype_IIDE_OL457490.1     ATGCAGTGGCGGCACCGAGTTCCTGGTGTCTGAGTGTATGGGAATCTGTTTCTGTCTCGC     239
                             ************************************************************

Genotype_IB_KY474061         TCGAAGACCCGATCGGCGCCCGTCGTCAACCCCC     274
Genotype_IIF_KX148658.1      TCGAAGACCCGATCGGCGCCCGTCGTCAACCCCC     274
Genotype_IA_KY474059.1       TCGAAGACCCGATCGGCGCCCGTCGTCAACCCCC     273
Genotype_IIDE_OL457490.1     TCGAAGACCCGATCGGCGCCCGTCGTCAACCCCC     273
                             **********************************
```

Supplementary Table 7. Bayesian model comparisons explaining *Ophidiomyces ophidiicola* prevalence across the landscape using the leave-one-out cross-validation (LOO).

**Model comparison tested**								
	elpd_diff	se_diff	elpd_loo	se_elpd_loo	p_loo	se_p_loo	looic	se_looic
Clade * Species + (1|Site)	0	0	-235.892	16.458	23.244	3.945	471.784	32.915
Clade + Species + (1|Site)	-0.163	3.165	-236.054	15.831	18.984	2.975	472.109	31.663
Species + (1|Site)	-0.759	3.571	-236.651	15.965	19.284	3.259	473.302	31.931
Clade + (1|Site)	-15.941	9.42	-251.833	14.088	11.754	1.082	503.666	28.176

Supplementary Table 8. Coefficients from Bayesian hierarchical model of interaction between species and clade explaining *Ophidiomyces ophidiicola* prevalence in Europe (coefficient ± standard deviation (95% credible intervals)). The data were analyzed with a binomial distribution which included site as a random effect, and species and clade as population-level effects. The model is parameterized in relation to the reference level or intercept *Natrix tessellata* and each parameter represents the difference from this reference level.

	Estimate ± Standard Deviation (95% CI)
Intercept Clade Europe: *N. tessellata*	-1.47±0.66 (-2.86,-0.21)
Clade North America: *N. tessellata*	1.15±0.85 (-0.58,2.82)
Species *N. helvetica*	0.8±1.22 (-1.54,3.46)
Species *N. natrix*	0.36±0.94 (-1.45,2.28)
Species *Z. longissimus*	-2.32±1.05 (-4.57,-0.45)
Clade North_America:species *N. helvetica*	-2.68±1.44 (-5.61,0.14)
Clade North_America:species *N. natrix*	-0.38±1.27 (-2.93,2.12)
Clade North_America:species *Z. longissimus*	1.6±1.29 (-0.74,4.34)

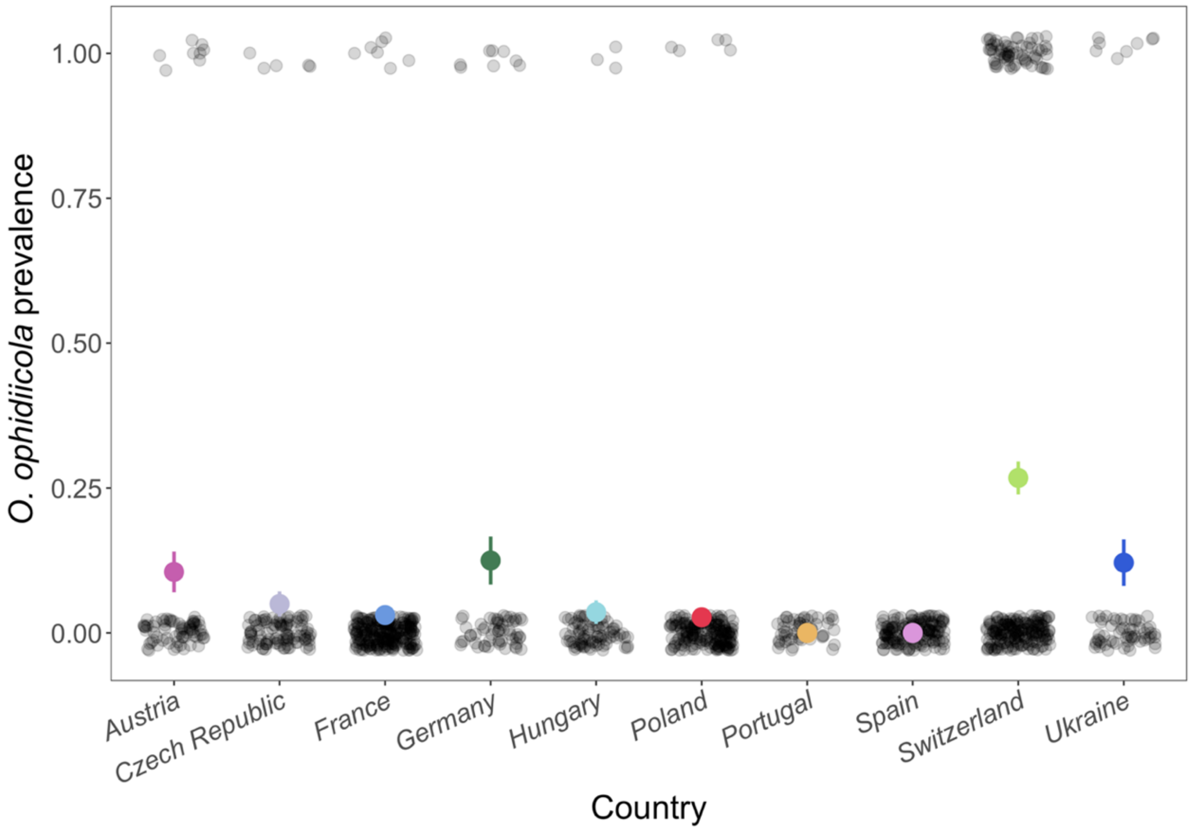

Supplementary Figure 1. Observed *Ophidiomyces ophidiicola* prevalence across all countries sampled using quantitative polymerase chain reaction (qPCR). Each transparent black point represents a single snake as being either negative (prevalence = 0) or positive (prevalence = 1). Color circles are mean prevalence ± standard error for each country.

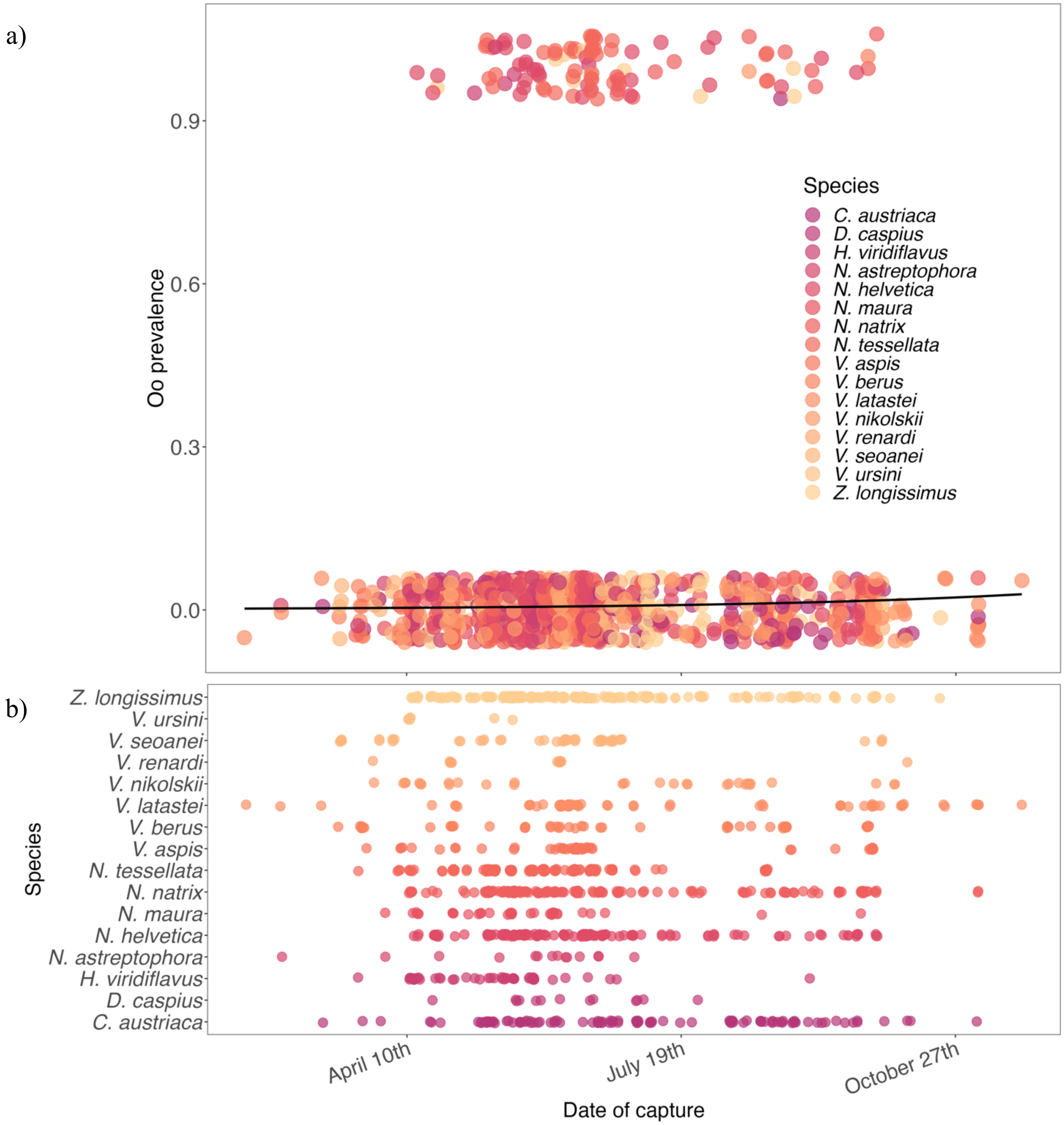

Supplementary Figure 2. Seasonality of *Ophidiomyces ophidiicola* prevalence and snake captures. a) Prevalence estimates over time. Color circles represent an individual detection (0|1) for a species, black line is the model fitted line b) and seasonality of species captures. Color circles represent an individual snake capture.

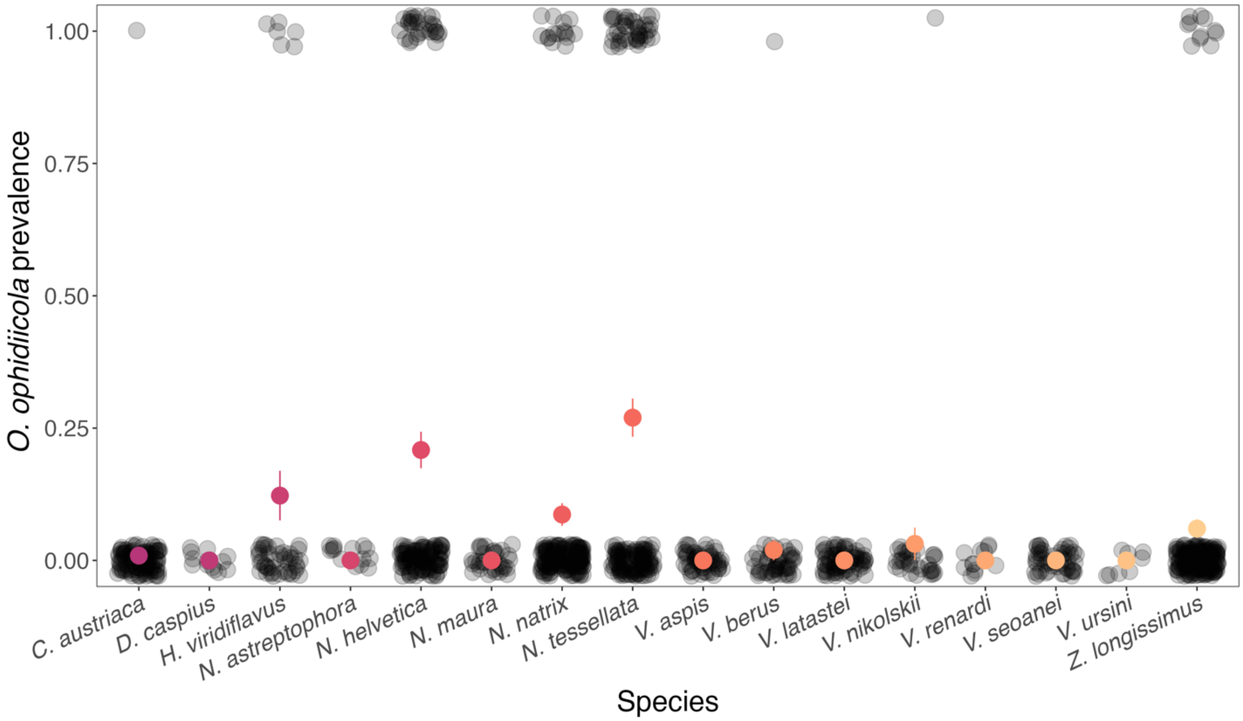

Supplementary Figure 3. Observed *Ophidiomyces ophidiicola* prevalence across all species sampled using quantitative polymerase chain reaction (qPCR), without the effect of site. Each transparent black point represents a single snake as being either negative (prevalence = 0) or positive (prevalence = 1). Color circles are mean prevalence ± standard error for each species.

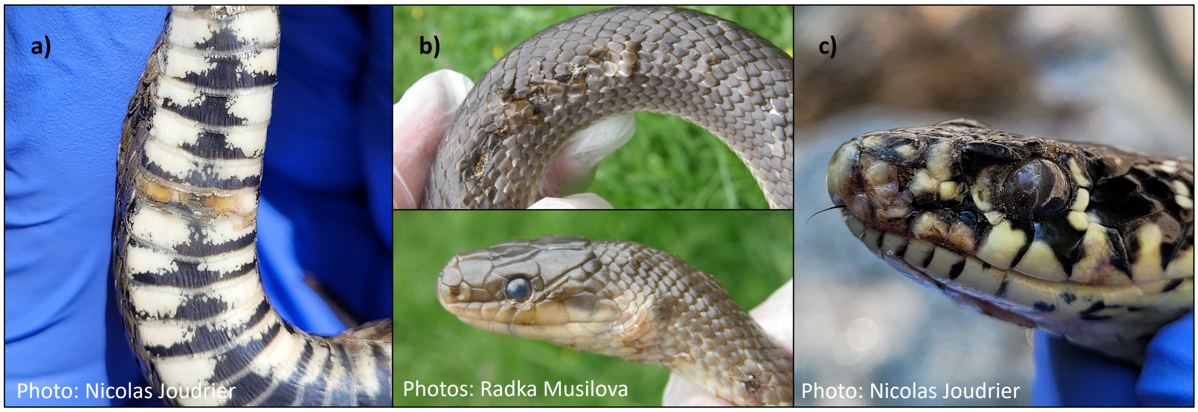

Supplementary Figure 4. Photos of lesions from snakes that were qPCR positive for *Ophidiomyces ophidiicola*. a) Photo of a ventral lesion on *Natrix tessellata* in Switzerland. b) Photo of lesions on the head and body of *Zamenis longissimus* in Czech Republic. c) Photo of a lesion on the head of *Hierophis viridiflavus* in Switzerland.

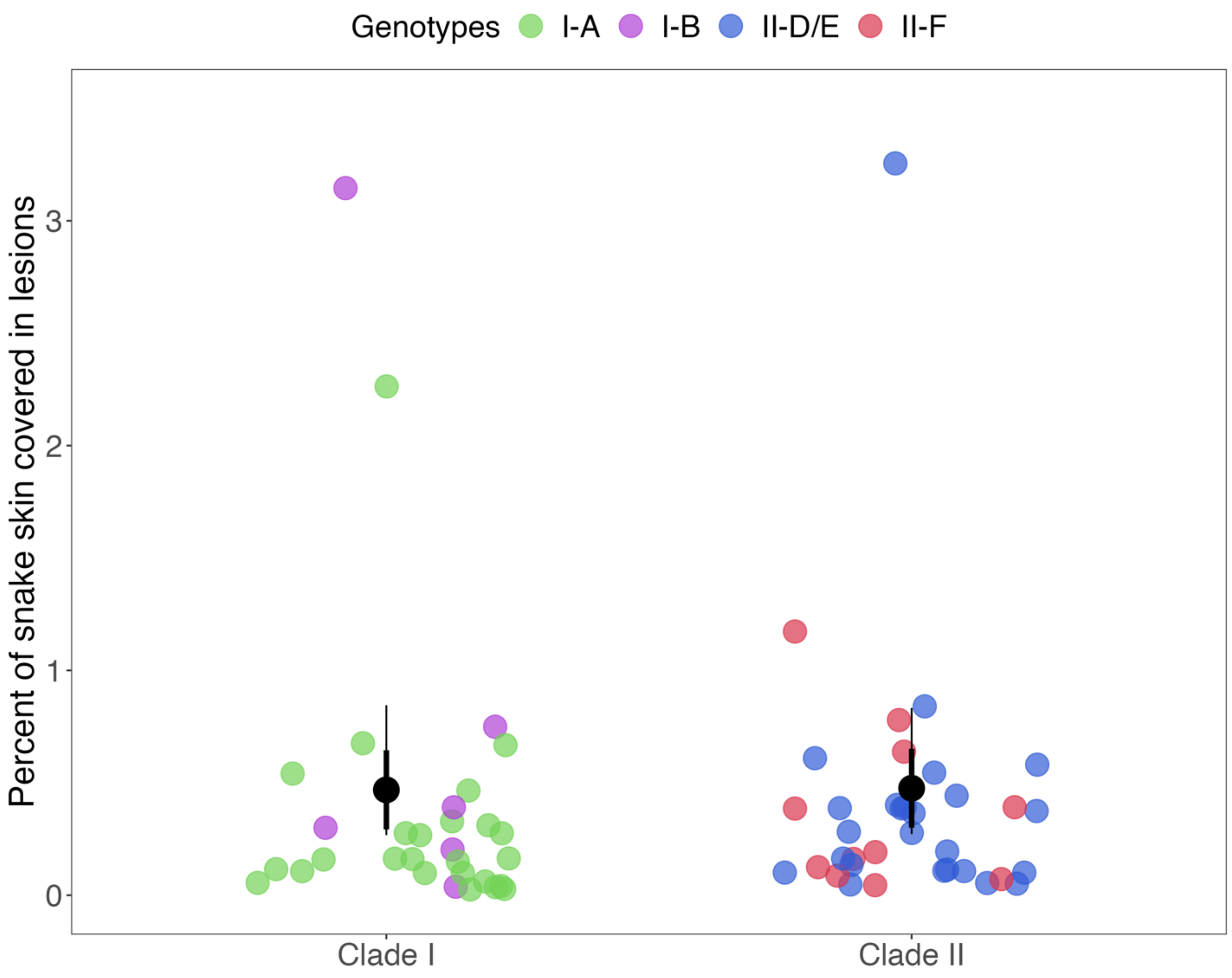

Supplementary Figure 5. Variation in disease severity (fraction of snake covered in lesions) by pathogen clade infecting the host. Each point is a single snake for which *Ophidiomyces ophidiicola* clade was determined, colors represent the different lineages of *O. ophidiicola*. The black circles and whiskers show the model predicted posterior mean, ± standard deviation (thick lines), and 95% credible intervals (thin lines).
