# Supplementary material for: Contribution of host species and pathogen clade to snake fungal disease hotspots in Europe
Source: Commun Biol. 2024 Apr 10;7:440. doi: 10.1038/s42003-024-06092-x (PMC11006896; doi:10.1038/s42003-024-06092-x)
Supplement: Supplementary file 5 — Reporting Summary [file 42003_2024_6092_MOESM5_ESM.pdf]

nature portfolio

Corresponding author(s): DAPR-23-4334

Last updated by author(s): Mar 11, 2024

Reporting Summary

Nature Portfolio wishes to improve the reproducibility of the work that we publish. This form provides structure for consistency and transparency in reporting. For further information on Nature Portfolio policies, see our Editorial Policies and the Editorial Policy Checklist.

Statistics

For all statistical analyses, confirm that the following items are present in the figure legend, table legend, main text, or Methods section.

n/a	Confirmed	
☐	☒	The exact sample size (*n*) for each experimental group/condition, given as a discrete number and unit of measurement
☐	☒	A statement on whether measurements were taken from distinct samples or whether the same sample was measured repeatedly
☐	☒	The statistical test(s) used AND whether they are one- or two-sided<br>*Only common tests should be described solely by name; describe more complex techniques in the Methods section.*
☐	☒	A description of all covariates tested
☒	☐	A description of any assumptions or corrections, such as tests of normality and adjustment for multiple comparisons
☐	☒	A full description of the statistical parameters including central tendency (e.g. means) or other basic estimates (e.g. regression coefficient) AND variation (e.g. standard deviation) or associated estimates of uncertainty (e.g. confidence intervals)
☐	☒	For null hypothesis testing, the test statistic (e.g. *F*, *t*, *r*) with confidence intervals, effect sizes, degrees of freedom and *P* value noted<br>*Give P values as exact values whenever suitable.*
☐	☒	For Bayesian analysis, information on the choice of priors and Markov chain Monte Carlo settings
☐	☒	For hierarchical and complex designs, identification of the appropriate level for tests and full reporting of outcomes
☒	☐	Estimates of effect sizes (e.g. Cohen's *d*, Pearson's *r*), indicating how they were calculated

Our web collection on statistics for biologists contains articles on many of the points above.

Software and code

Policy information about availability of computer code

Data collection	No software was used
Data analysis	Data was analyzed using the latest version of R 4.3.2

For manuscripts utilizing custom algorithms or software that are central to the research but not yet described in published literature, software must be made available to editors and reviewers. We strongly encourage code deposition in a community repository (e.g. GitHub). See the Nature Portfolio guidelines for submitting code & software for further information.

Data

Policy information about availability of data

All manuscripts must include a data availability statement. This statement should provide the following information, where applicable:

- Accession codes, unique identifiers, or web links for publicly available datasets
- A description of any restrictions on data availability
- For clinical datasets or third party data, please ensure that the statement adheres to our policy

All data are included in the article and supporting information. The code generated for this study will be made available in Dryad Digital Repository upon final acceptance.

Research involving human participants, their data, or biological material

Policy information about studies with human participants or human data. See also policy information about sex, gender (identity/presentation), and sexual orientation and race, ethnicity and racism.

Reporting on sex and gender	NA
Reporting on race, ethnicity, or other socially relevant groupings	NA
Population characteristics	NA
Recruitment	NA
Ethics oversight	NA

Note that full information on the approval of the study protocol must also be provided in the manuscript.

Field-specific reporting

Please select the one below that is the best fit for your research. If you are not sure, read the appropriate sections before making your selection.

☐ Life sciences ☐ Behavioural & social sciences ☒ Ecological, evolutionary & environmental sciences

For a reference copy of the document with all sections, see nature.com/documents/nr-reporting-summary-flat.pdf

Ecological, evolutionary & environmental sciences study design

All studies must disclose on these points even when the disclosure is negative.

Study description	Our study investigates the macroecological patterns of SFD across Europe by examining host and pathogen factors that may influence pathogen prevalence and disease severity across the landscape. We captured 1254 individual snakes from 21 species representing 6 genera in 10 countries, and collected a total of 2628 swabs which were analyzed by qPCR. Samples that were qPCR positive were then sequenced to determine which strain of the pathogen was responsible for the infection. We calculated SFD prevalence (# positives/# samples) for each site and species and reported 95% confidence intervals. We evaluated the presence of hotspots, the difference in prevalence across host species species and the effect of both host species and pathogen genotype on disease prevalence using Bayesian hierarchical models reporting mean +/- SD and confidence intervals. All models tested are reported in a supplemental table which describes the statistical analysis and the parameters of the models.
Research sample	We collected samples from 21 different snake species. We were generally interested in sampling all species present in each region being sampled. We did not restrict our sampling to any specific sex or age range and our sampling generally represents the snake community present at the time of data collection.
Sampling strategy	At each site, we aimed for a minimum of 4 species and ~10 individuals from each species based on power analyses.
Data collection	Data and samples were recorded by an observer in the field. Snakes were swabbed in duplicate (except for a few individuals that were swabbed only once due to limitations in the field) using a pre-moistened, sterile polyester-tipped applicator by running the swab five times (back and forth counting as a single pass) on the ventral and dorsal areas (from the neck down to the vent), and two times on the face of the snake. If a skin lesion was observed, a separate swab was used to specifically swab the lesion and skin immediately adjacent to it by rubbing the swab over the affected skin.
Timing and spatial scale	Snakes were captured from March 2020 to June 2022 across 10 countries: Portugal, Spain, France, Switzerland, Germany, Austria, Czech Republic, Hungary, Poland, and Ukraine. The number of sites where snakes were collected ranged from two to eleven per country, for a total of 61 sites. Sample sizes were the highest in Spring season due to the seasonal behavior of snakes who came out of hibernation in Spring and start foraging and mating.
Data exclusions	For all analyses, we excluded any species and sites that had been sampled fewer than eight times, because of the high uncertainty of the model predictions under this threshold.
Reproducibility	All snakes were swabbed in duplicate or triplicate, and each swab was analyzed in duplicate. We used negative controls for both the extraction and qPCR steps
Randomization	We were restricted in where we could sample due to local/regional permitting requirements. In general, sites were selected based on pre-existing knowledge of snake presence or prediction of suitable habitats to sample as many species and individuals as possible. All other grouping was done by species so no randomization is required.
Blinding	Individuals testing the samples were blind to the condition and location of the animals sampled.

Did the study involve field work? ☒ Yes ☐ No

Field work, collection and transport

Field conditions	Study conditions were highly variable based on location and time of year. No field work was conducted in rainy condition or when temperature was below 10C (average temperature across all sampling location = 24.4C), as snakes would be under cover or in hibernation. All animals were processed in the field where they were captured and released at the exact location of capture.
Location	Austria: in region Lower Austria, Vienna and Styria (site lat/lon ranges: Aus1 48.18-48.15 / 16.49-16.55, Aus2 48.04-47.80 / 16.08-16.22, Aus3 48.71-48.72 / 15.65-15.66, Aus4 48.15-48.14 / 16.68-16.70, Aus5 47.38-47.25 / 14.92-15.16)<br>Czech Republic: in region Karlovary Vary (site lat/lon ranges: Cz1 50.39-50.34 / 13.28-13.37, Cz2 50.43-50.32 / 12.82-13.08)<br>France: France-Comte and Loire-Atlantique regions (site lat/lon ranges: Fr1 47.78-47.38 -1.87/-1.67, Fr2 47.77-47.59 / 6.39-6.76, Fr3 47.15-47.36 / 6.68-6.87, Fr4 47.32-47.27 / 6.12-6.27, Fr5 47.20-47.09 / 6.44-6.72, Fr6 46.61-46.47 / 5.70-6.02, Fr7 46.34-46.24 / 5.52-5.68, Fr8 47.25-46.81 / 5.65-6.24)<br>Germany: Upper/Lower Bavaria (site lat/lon ranges: Ger1 48.66-48.49 / 13.39-13.76, Ger2 48.30-48.14 / 12.71-13.08, Ger3 47.84-47.61 / 11.98-12.47, Ger4 48.20-48.12/11.30-11.51, Ger5 47.73-47.58/11.08-11.30, Ger6 48.03-47.76/11.22-11.45)<br>Hungary: Bacs-Kiskun, Borsod-Abauj-Zemplen, Budapest, Gyor-Moson-Sopron, Heves<br>Nograd, Pest, Somogy, Vas (site lat/lon ranges: Hun1 48.29-48.14/21.25-21.54, Hun2 48.09-47.99/18.85-19.09, Hun3 47.14-46.94/19.18-19.52, Hun4 47.60-47.41/18.96-19.31, Hun5 47.85-47.60/17.14-17.54, Hun6 47.42-47.20/17.07-17.41, Hun7 46.40-46.20/17.06-17.38)<br>Poland: Kuyavia-Pomerania, Lesser Poland, Lower Silesia, Opole, Silesia, Subcarpathia (site lat/lon ranges: Pol1 53.15-52.95/17.78-18.20, Pol2 51.44-51.30/15.46-15.80, Pol3 51.25-51.05/16.86-17.25, Pol4 50.59-50.44/17.90-18.23, Pol5 49.87-49.72/18.43-18.78 ,Pol6 50.47-50.02/21.20-21.82, Pol7 50.11-49.95/19.77-20.12, Pol8 49.32-49.14/22.35-22.80)<br>Portugal: Portalegre (Alentejo region) and Braga/porto/Viana do Castelo (region Norte), site lat/lon ranges: Por1 42.05-41.28 -8.73/-8.12, Por2 38.87-38.68 -7.30/-7.01)<br>Spain: Huelva (Andalusia), Leon/Zamora/Burgos (Castile y Leon), Galicia, La Rioja, Navarra ((site lat/lon ranges: Sp1 43.70-41.98 -8.86/-7.27, Sp2 43.07-42.17 -6.72/-3.33, Sp3 37.26-36.82 -6.95/-6.26, Sp4 42.49-42.11 -2.98/-2.21, Sp5 43.03-42.83 -1.73/-1.39)<br>Switzerland: Bern, Neuchatel, Nidwalden, Obwalden, Schwyz, Ticino, Vaud (site lat/lon ranges: Sw1 46.28-45.84 8.53-8.98, Sw2 46.99-47.21 8.57-8.80, Sw3 46.98-46.94 8.27-8.33, Sw4 46.79-46.68 8.19-8.36, Sw5 46.75-46.67 7.89-8.07, Sw6 46.36-46.20 6.87-7.23, Sw7 46.50-46.39 6.12-6.29, Sw8 46.55-46.44 6.66-6.92, Sw9 46.90-46.74 6.53-6.75, Sw10 47.03-46.94 6.67-6.85, Sw11 47.04-46.95 6.95-7.11)<br>Ukraine: regions Chernihiv, Crimea, Kharkiv, Kherson, Luhansk, Mykolaiv, Odesa (site lat/lon ranges: Ukr1 51.22-51.02 30.58-30.98, Ukr2 47.89-47.73 29.30-29.59, Ukr3 46.72-46.60 31.03-31.27, Ukr4 47.26-47.06 31.89-32.14, Ukr5 46.37-46.07 33.13-33.49, Ukr6, 46.48-46.31 35.10-35.37, Ukr7 50.18-49.29 35.12-37.09)
Access & import/export	Local and international regulations were followed at all times. Handling of snakes was reviewed and approved by Virginia Tech Institute for Animal Care and Use Committee protocol 20-055. Permits to conduct our field study were obtained when necessary, and were granted by the Regional office of the Karlovarian region, Department of Environment and Agriculture in Czech Republic (permit # KK/1098/ZZ/20-4), the General and Regional Directorates of Nature Conservation in Poland (permit # DZP- WG.6401.91.2020.TŁ; DZP-WG.6401.91.2020.TŁ.2, WPN.6401.270.2019.MF, WPN.6401.17.2020.KW.2, WPN.6401.9.2021.KW.2), Portuguese and Spanish wildlife legislation (permits # 295/2020/CAPT, 146/2021/CAPT, 201999902471003/IRM/MDCG/mes, EB-018/2020, EB-015/2021, AUES/CYL/192/2020, AUES/CYL/54/2021, A/2021/036, 0001-0261-2021-000003), the Direction régionale de l'environnement of Franche-Comte (ONAGRE 2020-01-17-00122; 25-2022-02-17-00001) and Loire-Atlantique (Cerfa 13616, permit # 64/2016) in France, the Museum of Natural History in Budapest, Hungary (permit # PE-KTFO/1568-18/2020), Austrian legislations (permits # MA22 – 1089768-2020, RU5-BE-64/023-2022, RU5-BE-64//022-2021, ABT13-53W-50/2018-2), the Federal Office for the Environment (FOEN) in Switzerland with veterinary authorization (VD3718, ID 33612), and cantonal authorizations (Neuchatel: FS-08/2021, Grisons: AV-2022-338, Vaud: 2021-3537).
Disturbance	Efforts were made to reduce disturbance to animals and field sites. Animals were released on site at the point of capture. Processing time for animals was ~15 min. per animal.

Reporting for specific materials, systems and methods

We require information from authors about some types of materials, experimental systems and methods used in many studies. Here, indicate whether each material, system or method listed is relevant to your study. If you are not sure if a list item applies to your research, read the appropriate section before selecting a response.

Materials & experimental systems

n/a	Involved in the study
☒	☐ Antibodies
☒	☐ Eukaryotic cell lines
☒	☐ Palaeontology and archaeology
☐	☒ Animals and other organisms
☒	☐ Clinical data
☒	☐ Dual use research of concern
☒	☐ Plants

Methods

n/a	Involved in the study
☒	☐ ChIP-seq
☒	☐ Flow cytometry
☒	☐ MRI-based neuroimaging

Animals and other research organisms

Policy information about studies involving animals; ARRIVE guidelines recommended for reporting animal research, and Sex and Gender in Research

Laboratory animals	NA
Wild animals	No animals were housed. All animals were captured in the field, processed at the field site, and immediately released at the location they were captured. We captured in total the following species (sample size in parentheses): Coronella austriaca (122), C. girondica (3), Dolichophis caspius (16), Elaphe sauromates (3), Hierophis viridiflavus (59), Malpolon monspessulanus (3), Natrix astreptophora (15), N. helvetica (145), N. maura (39), N. natrix (176), N. tessellata (162), Vipera ammodytes (7), V. aspis (72), V. berus (62), V. latastei (75), V. nikolskii (32), V. renardi (12), V. seoanei (51), V. ursini (14), Zamenis longissimus (183), Z. scalaris (3)
Reporting on sex	Data on the sex of each animals was determined based on morphology or after probing. Multiple studies have shown no effect of sex on disease status, therefore, both sexes were combined for analyses.
Field-collected samples	Snakes were captured and placed individually in cloth or disposable paper bags for a maximum of 10 minutes before being processed, which minimized stress. Bags were placed in the shade to avoid direct sun exposure. We then collected skin swabs for all individuals, measured and weighted the snakes, and measured and took photos of skin lesions when present on the body. Snakes were then released at the point of capture.
Ethics oversight	Protocols were approved under Virginia Tech IACUC protocol #20-055

Note that full information on the approval of the study protocol must also be provided in the manuscript.

Plants

Seed stocks	NA
Novel plant genotypes	NA
Authentication	NA
